# A Novel Adjuvant to the Resident Selection Process: the Hartman Value Profile

**Published:** 2012-06-13

**Authors:** Jeffrey D. Cone, C. Stephen Byrum, Wyatt G. Payne, David J. Smith

**Affiliations:** ^a^Division of Plastic Surgery, Department of Surgery, University of South Florida, Tampa; ^b^The Byrum Consulting Group, LLC, Signal Mountain, TN; ^c^Plastic Surgery Section, Bay Pines VA Healthcare System, Bay Pines, FL

## Abstract

**Objectives:** The goal of resident selection is twofold: (1) select candidates who will be successful residents and eventually successful practitioners and (2) avoid selecting candidates who will be unsuccessful residents and/or eventually unsuccessful practitioners. Traditional tools used to select residents have well-known limitations. The Hartman Value Profile (HVP) is a proven adjuvant tool to predicting future performance in candidates for advanced positions in the corporate setting. **Methods:** No literature exists to indicate use of the HVP for resident selection. **Results:** The HVP evaluates the structure and the dynamics of an individual value system. Given the potential impact, we implemented its use beginning in 2007 as an adjuvant tool to the traditional selection process. **Conclusions:** Experience gained from incorporating the HVP into the residency selection process suggests that it may add objectivity and refinement in predicting resident performance. Further evaluation is warranted with longer follow-up times.

The dilemma of recruiting and appointing Plastic Surgery residents is this: how does one predict performance of a resident and practitioner on the basis of past performance in medical school? Current selection processes attempt to answer this question by relying on applicant resumes, letters of recommendation, personal discussions and interviews. This information is either relatively objective, as is the case for United States Medical Licensing Exam (USMLE) scores, Alpha Omega Alpha Honor Society (AOA) membership, and class rank, or subjective, as is the case for letters of recommendation, medical school reputation, and clerkship performance. Programs assign varying weight to each piece of information and then rank applicants on the basis of their overall thoughts, experience, and “general gestalt.”^1^^-^5

The flaw of this design is obvious. Actual performance of a resident, and an eventual plastic surgeon, is based upon his or her ability to execute sound judgments within the complex setting of health care. This ability relates directly to character qualities such as intelligence, integrity, adaptability, maturity, leadership, and work ethic.^1^^-^3^,^^5^^,^^6^ Although numerous publications on the subject of resident selection give credence to the critical nature of these qualities, none directly evaluate these qualities or their implications on performance.^1^^-^3^,^^5^^,^^6^

The Hartman Value Profile (HVP) is an effective, proven methodology for the prediction of performance.^7^^,^^8^ Because it specifically addresses the aforementioned character qualities, it is utilized extensively in private industry for employee selection and development. Its predictive value in advanced medical employment, and specifically, in choosing eventual, successful candidates for residency programs is untested.

No literature exists to indicate its use for resident selection. Given the potential advantages, we implemented its usage beginning in 2007 as an adjuvant tool to the traditional selection process. This article outlines what the HVP is, details its potential impact as an adjuvant to the selection process, and specifies how it might contribute objectivity to the research of resident selection and training.

## ABOUT THE HARTMAN VALUE PROFILE

The HVP is based upon formal axiology, a field of psychology that evaluates how individuals assign value to themselves and to the surrounding environment. It is not an IQ/rational intelligence profile, a personality test (like the Myers-Briggs), or an emotional balance profile (like the Minnesota Multiphasic Personality Inventory). In fact, it demonstrates the limitations of each of these (for instance, the fact that someone can lack “common sense” despite being “book smart,” or that individuals can have a major personality flaw and yet be highly effective professionals).

Instead, it evaluates the structure and the dynamics of an individual's value system. Dr Robert S. Hartman, for whom the profile is named, argued that value is assigned to various concepts or objects according to the following:

1. The value of its uniqueness (described as “Intrinsic Value Dimension”)
2. The value of its function or role (or “Extrinsic Value Dimension”)
3. The value of its meaning or purpose (or “Systemic Value Dimension”)

The Intrinsic Value Dimension evaluates the capacity for relational judgment, which is evidenced simply in good people skills. The Extrinsic Value Dimension involves the capacity for excellence in tasks, projects, processes, and the basic implementation of skill competencies. The Systemic Value Dimension reveals the capacity for excellence in long range planning, strategic visioning, structural integrations, implications, and consequences.

Furthermore, each of these dimensions can be valued or de-valued intrinsically, extrinsically, or systematically. For example, if a person admires a specific car, then he or she intrinsically values the intrinsic value of the car. If the person enjoys driving a specific car, then he or she extrinsically values the intrinsic value of the car. If the person thinks the uniqueness of the car is absurd or crazy, then he or she systemically devalues the intrinsic value of the car. By combining the 6 variations of value judgments for intrinsic, extrinsic, and systemic dimensions, a total of 18 value judgments can be made.

Axiology demonstrates that these 18 possibilities are not assigned randomly; instead, the relative value that an individual assigns to an object, a choice, or a circumstance is based upon that individual's conceptual system or hierarchy. In other words, people interpret a circumstance, evaluate it according to previous experiences, and then make decisions accordingly. Since this process is the basis for the practice of medicine, evaluation of this system/hierarchy would be an important component of selection of Plastic Surgery resident candidates.

Individuals employ this hierarchical arrangement of value with regularity and use their personal value systems as compasses to navigate daily circumstances and choices. The HVP details this conceptual system and therefore lends insight into how people view themselves, others, and the world around them.

*Evaluative judgment* is defined as the ability, when presented an issue or problem or situation, to observe and understand the dynamics of the situation, to determine what actions will make the situation better, and ultimately take action to improve the situation. Evaluative judgments involve 3 general levels, and each lends significant insight into how individuals navigate daily interactions.

The first, intrinsic judgment, demonstrates the ability to be savvy about others and evaluate other individuals in a discerning manner. The second, extrinsic judgment, is task or work judgment and relates to performing a job with effectiveness, efficiency, and dependability. The third, systemic judgment or big-picture judgment, relates to the ability to understand implications and consequences.

These results exist within a spectrum from very weak to very strong. They are numerical and include global scores and scores of the various components. Table 1 lists the components of self-side and work-side judgment. Table 2 details the balance indicators of work-side judgment and self-side judgment. The “Self-side” addresses how participants value themselves (eg, the degree to which they possess self-confidence, assertiveness, and adaptability). The “work-side” addresses how they value their work (eg, the degree to which they possess trainability and dependability). Extensive validation studies confirm that the profiles are not biased to race, age, sex, or ethnicity, and are highly reliable and reproducible.^7^

Accordingly, Biderman et al^8^ found a positive correlation between HVP scores and undergraduate student performance. Drs Smith and Harvey found a 90% successful rate for managerial candidates with a low level of risk before employment as determined by the HVP.^7^ Follow-up studies with the Sara Lee Corporation and with the James River Corporation showed that the HVP predicted exemplary performance in managerial success and customer service, respectively.^7^ The first of these studies defined success as having been commended by clients and/or colleagues for exemplary customer service. The second study distinguished excellence according to operations, sales, low position turnover, and the ability to function within budget; all well-known measures of business success. These 2 corporations emphasized the components of the HVP that deal with empathy, healthy self-esteem, reasonability, and the ability to delegate, in their selection process for these managerial positions.

## INTEGRATING THE HARTMAN VALUE PROFILES INTO THE RESIDENT SELECTION PROCESS

**Figure 1:**
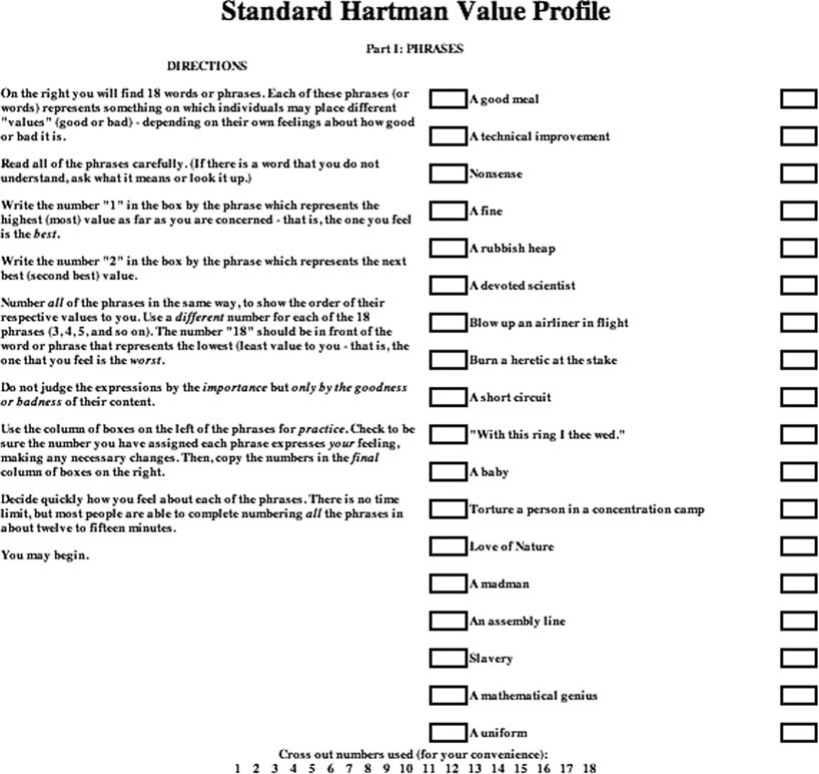
“Part I: Phrases”—Value judgment ranking list as the individual relates primarily to the world of work (or the world that is “external”).

**Figure 2:**
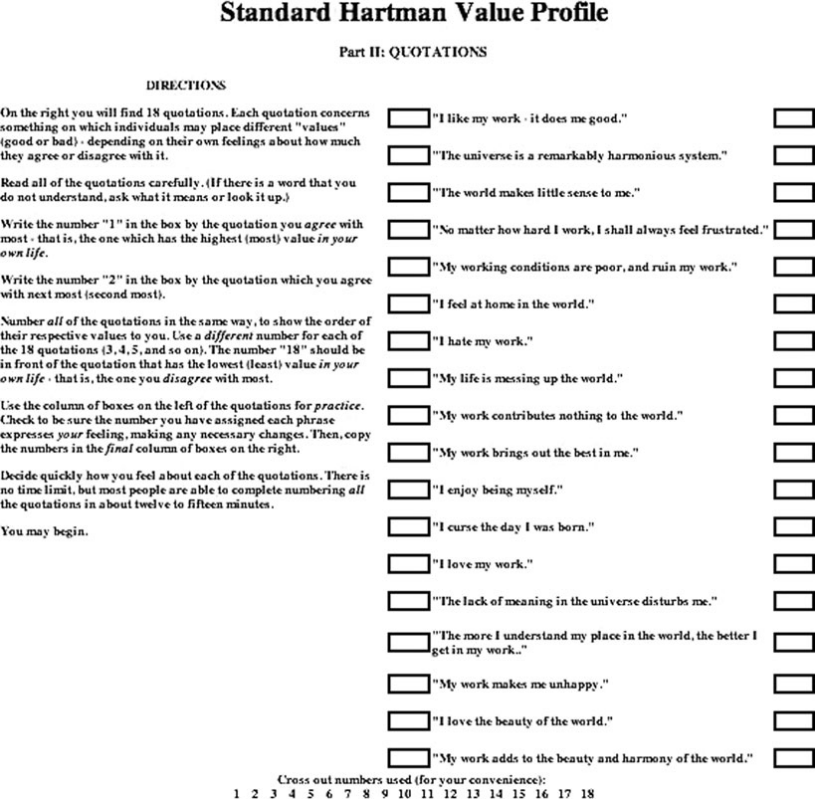
“Part 2: Quotations”—Value judgment ranking lists as it pertains to the individual's judgments concerning one's self (or to the “internal” self).

Individualized HVPs are generated from the manner by which subjects rank 2 lists of 18 phrases. The first list is ranked from “best to worst” (Fig 1) and the second from “most agree to least agree” (Fig 2).^9^ Before completing the forms, applicants are asked to read the standard instructions printed beside the phrases. The profiles reflect individual preference; accordingly, it is emphasized that no right or wrong answers exist and that honesty is the best criterion for obtaining accurate results.

The profiles take approximately 15 to 25 minutes to complete. Most applicants finish in one sitting between interview sessions. Each applicant's responses are collected at the end of the interview day.

The HVP of the applicants are interpreted with blinding as to the individual's name and demographics. The results are derived from logical mathematical norms with numerical values for each of the components listed in Tables 1 and 2. There are several consulting groups, which implement and interpret the HVP.^7^^,^^9^ Our experience is that a consultant is desirable for customizing the HVP to the needs of a specific institution, for specific desired effects/outcomes/emphasis. However, individuals within an institution can be trained to interpret the profiles and thus avoid dependency on consultants. The costs are relatively low with either approach.

Because the results of the HVP include global scores and a myriad of component scores, it should be noted that the tool is almost infinitely adaptable for the uniqueness of individual environments. Programs may differ on the characteristics they are seeking, and specific components of the HVP can be emphasized to target candidates who best fit a program's personality and goals.

## DISCUSSION

The primary goal of resident selection process is to identify which residency candidates will mature into successful residents and then into successful practitioners. The degree to which this goal is achieved has immediate and long-ranging impacts. On one level, these residents play a critical role in defining the day-to-day, operational fluidity of a residency program and its ability to provide competent patient care. On a higher level, these residents ultimately shape the future of the field of Plastic Surgery. Individual programs have specific individual, unique needs or requirements that may differ from other programs.

Currently, the selection process attempts to achieve this goal by analyzing information made available through the match process, including applications, letters of recommendation, and interviews. Programs assign various weights to these pieces of information, and rank applicants on the basis of the impressions generated by this relatively subjective and unstandardized process.^1^^-^5

This design is flawed, and the literature regarding the selection processes for Plastic Surgery residents reveals several limitations. Janis and Hatef^2^ reported that only 45.2% of respondent program directors found the current process to be successful in identifying potential problems before matriculation. Moreover, attrition rates are unacceptably high: one-third had a resident resign within the past 10 years and two-fifths dismissed a resident within the past 10 years for academic or ethical reasons.^2^ Other specialties echo this experience. For instance, program directors in orthopedic residency reported that 1 in 6 resident selections was thought to be inappropriate and 1 in 12 was considered a serious mistake.^3^^,^^5^

The lasting effects of resident selection are significant. From a program's perspective, a danger exists in hiring a candidate who proves to be unsuccessful as a resident (judged by disciplinary actions, poor evaluations or by attrition), and/or as a practitioner (judged by revocation of board certification or licensure, or the existence of felonies or crimes). From a candidate's perspective, a danger exists of being overlooked because of incorrect inferences from an application, a letter of recommendation, or interview. Dissatisfaction exists, and every publication on the subject calls for refinement.^1^^-^6^,^^10^^-^13

Perhaps more telling than the conclusions of the studies are the designs of the studies themselves. Past literature on the subject is directed either toward (1) correlating curriculum vitae (CV) components to future resident or practitioner success^4^^,^^6^^,^^11^^,^^13^^-^18 or (2) presenting cross-sectional analysis of how residency programs assign weight to CV components and interviews.^1^^-^3

Clearly, current evaluation processes provide important insight and have made significant contributions to the candidate assessment and resident selection process. Yet, a number of limitations exist.

The first is the fact that most of the data are *subjective*, including letters of recommendation, school reputation, interview performance, publications, appearance, and dean's letter.^3^^-^5 Although a characteristic or interaction with an applicant can generate a unique and strong impression, it is often impossible to define why the impression was so positive (or negative), to measure the extent to which it was positive (or negative) and then apply that impression to future candidate selections.

For example, Gladwell^19^ outlines the tendency of interviewers to fixate on supposedly stable character traits and overlook the influence of context. This phenomenon is referred to as the *Fundamental Attribution Error* and leads to conclusions that “the most basic of human rituals—the conversation with a stranger—turns out to be a minefield.”^19^

Authors have attempted to overcome this deficit by generating composite scores.^11^ Yet, metrics cannot regularly and reliably be applied to subjective values without adopting a variable degree of error. For example, AOA membership is an apparently binary qualification; either a candidate is a member (denoted by a 1) or not (denoted by a 0). Yet, AOA membership is awarded according to different criteria at different institutions, and some involve a component of peer election. When this is taken into account, a seemingly binary qualification is splintered into subclassifications. Objective, independent confirmation of the impressions generated by the selection process is often elusive, and this lack of confirmation restricts research on the field.

Insight into the degree of importance assigned to which components of applicant's CV and interview has been discussed extensively.^1^^-^3 Yet, at their core, these studies are surveys, and as such, possess the inherent limitation of being subjective reports regarding relatively subjective data. For example, a program director may report that his or her program emphasizes letters of recommendations above every other component of the application. It remains impossible to determine (1) whether this statement is true and 2) to what degree is it true. The validation of these data, which is a key to the data's usefulness, is lacking.

Descriptions of selection processes provide valuable but limited information. The inability to significantly refine the resident selection process is due in part to the limited correlation of applicants' CVs and interviews to those traits, which are key to resident success. Publications affirm that successful residents are defined by the traits of “honesty, integrity, and a good work ethic,” and note that insight into these traits would improve the selection process.^1^^,^^3^^,^^5^^,^^12^ Adaptability and the ability to organize one's thought process are considered defining qualities of competency in residency.^2^ A lack of a “teamwork” mentality in recent graduates may explain limitations in coverage for emergency room, referrals, and patients.^6^

Positive qualities are certainly desirable in any/all individuals, and especially in candidates considered for inclusion in Plastic Surgery Residency Programs. Again, problems in determining, confirming, and assessing these qualities in individuals considered for residency positions exist, because independent, objective confirmation is difficult to attain in the absence of a value assessment tool.^3^^,^^6^^,^^12^^,^^20^

On one hand, a call to improve the selection process exists. On the other hand, an emphasis on the critical nature of character qualities exists.^1^^-^3^,^^6^^,^^12^ Accordingly, we suggest that usage of the HVP as an additional tool in resident selection may provide significant refinement by lending insight into these qualities, and ultimately into the ability of a candidate to make sound judgments as he or she navigates the complex setting of health care. The HVP provides objectivity to a previously subjective process, and more closely links the application process to the goal of the selection process.

Our initial experience in utilizing the HVP as an adjuvant tool to the selection process has been positive. The experience, albeit anecdotal, suggests early findings. Over the period of its use, the program's attrition rate has been zero and the frequency of faculty and resident complaints involving residents appears to have declined. Though numbers to date have been relatively small (15 individuals over a 5-year period), and long-term outcome data are absent, interim evaluations and mid-term HVP assessments appear to indicate a positive correlation with resident performance.

## FUTURE APPLICATIONS

To gain insight into the training process in general, we retested 9 residents at the end of their Post Graduate Year 3 (which represents the mid-way point of our 6-year program). From a pedagogical perspective, residency is a form of training, and “training” is distinct from “education” because it entails more than the acquisition of information. It entails the acquisition of a skill set *and* the decision process to apply this skill set.

The HVP has proven beneficial in the way that it has captured the attention of residents in debriefing sessions at the mid-way point of residency. It presents both affirmation of strengths and awareness of areas for needed development of stronger judgment. By offering the tool at intervals in the residency program, there is additional evidence gained on the impact—both positively and negatively—of the overall program's influence on the evolving of resident judgment. Of critical importance has been those indicators on the profile that relate to stress, because stress can be the primary obstacle to and “de-railer” of optimal judgment. Evidence confirms a positive correlation of these indicators to predicting future success.^8^ Accordingly, we intend to use the HVP in evaluating the training process, because it may provide objective data on items such as stress management ability and overall progress.

Although axiology demonstrates that value systems are consistent across concepts, it also demonstrates that these systems are dynamic over time. Value systems are a product of interactions, events, and relationships, and additional experiences will refine one's value system. For example, if a person who is routinely disorganized consistently emphasizes habits that created organization, then he or she could become organized over time.

This carries special significance in light of the core competencies provided by The Accreditation Council for Graduate Medical Education (ACGME). According to the ACGME, these core competencies constitute the cornerstone of medical education.^21^ Yet, as Dumanian^22^ detailed, these competencies lack evidence demonstrating that they improve the education process. The HVP can lend specific insight into these competencies and could challenge assumptions regarding how residency training addresses the core competencies. For instance, how do residency programs impact stress management, organizational skills, trustworthiness, confidence, communication skills, or passion for one's profession?

## CONCLUSIONS

The goal of the resident selection process is twofold: (1) to select candidates who will be successful residents and eventually successful practitioners and (2) to avoid selecting candidates who will be unsuccessful residents and/or eventually unsuccessful practitioners. Traditional tools used to select residents have well-known limitations. The HVP is a proven adjuvant tool to predict future success in candidates in the corporate setting. Our experience gained from incorporating the HVP into the residency selection process suggests that it may add objectivity and refinement in predicting resident performance. Further study utilizing multiple institutions and medium- and long-term outcomes are necessary.

## Acknowledgments

This study was not supported by funds or grants. The contents of this report do not represent the views of the Department of Veterans Affairs or the US government. Dr Byrum serves as the president for the Byrum Consulting Group, which implements the Hartman Value Profiles as part of their corporate consultations. None of the authors received payments or benefits related to this article.

## Figures and Tables

**Table 1.** Measured components of self- and work-side judgment

	Personal/Self-Side Judgment	External/Work-Side Judgment
1.	Understanding what is “important”	Problem solving ability
2.	Self-regard/self-care	Ability to notice, insight, sensitivities
3.	Self-accepting vs self-criticizing	Dealing with difficult situations, problem solving energy, innovation
4.	Effects of self-side stress	Effects of work-side stress
5.	Assertive vs conflict avoidant	Focus and concentration
6.	Moral clarity	Directions followed with accuracy
7.	Problem solving style, self-side	Problem solving style, work-side
8.	Acceptance of change/role identity	Realisms vs idealism orientation
9.	Meaningfulness of work, self-identity	General tolerance, acceptance of others
10.	Morale: value of work	Compassion, empathy, actions of care
11.	Solving personal problems for self	Trainability- the ability to understand work
12.	Solving practical problems for self	Dependability, reliability, work ethic
13.	Basic organizational ability	Understanding big picture implications
14.	Environmental conscientiousness	Using big picture implications
15.	Overall strength of self-side judgment	Overall strength of work-side judgment

**Table 2:** Balance indicators of self- and work-side judgment

	Self-Side Balance	Work-Side Balance
1.	Self-esteem/self-confidence	Value of people, relations
2.	Self-confidence/Role of Satisfaction	Value of work, tasks
3.	Self-image/Motivation	Value of ideas, implications and consequences
